# The factor structures and correlates of PTSD in post-conflict Timor-Leste: an analysis of the Harvard Trauma Questionnaire

**DOI:** 10.1186/s12888-017-1340-0

**Published:** 2017-05-22

**Authors:** Alvin Kuowei Tay, Mohammed Mohsin, Susan Rees, Zachary Steel, Natalino Tam, Zelia Soares, Jessica Baker, Derrick Silove

**Affiliations:** 1 Psychiatry Research and Teaching Unit, Academic Mental Health Unit, School of Psychiatry, University of New South Wales, Cnr Forbes and Campbell Streets, Liverpool, NSW 2170 Australia; 2 The Black Dog Institute, Sydney, Australia; 3 St John of God, Richmond Hospital, Richmond, NSW Australia

**Keywords:** Harvard Trauma Questionnaire, PTSD, ICD-10, ICD-11, DSM-IV, Emotional numbing, Dysphoric-arousal, Trauma

## Abstract

**Background:**

Post-traumatic stress disorder (PTSD) is the most widely assessed form of mental distress in cross-cultural studies conducted amongst populations exposed to mass conflict and displacement. Nevertheless, there have been longstanding concerns about the universality of PTSD as a diagnostic category when applied across cultures. One approach to examining this question is to assess whether the same factor structure can be identified in culturally diverse populations as has been described in populations of western societies. We examine this issue based on an analysis of the Harvard Trauma Questionnaire (HTQ) completed by a large community sample in conflict-affected Timor-Leste.

**Method:**

Culturally adapted measures were applied to assess exposure to conflict-related traumatic events (TEs), ongoing adversities, symptoms of PTSD and psychological distress, and functional impairment amongst a large population sample (*n* = 2964, response rate: 82.4%) in post-conflict Timor-Leste.

**Results:**

Confirmatory factor analyses of the ICD-10, ICD-11, DSM-IV, four-factor Emotional Numbing and five-factor Dysphoric-Arousal PTSD structures, found considerable support for all these models. Based on these classifications, concurrent validity was indicated by logistic regression analyses which showed that being a woman, trauma exposure, ongoing adversity, severe distress, and functional impairment were all associated with PTSD.

**Conclusions:**

Although symptom prevalence estimates varied widely based on different classifications, our study found a general agreement in PTSD assignments across contemporary diagnostic systems in a large conflict-affected population in Timor-Leste. Further studies are needed, however, to establish the construct and concurrent validity of PTSD in other cultures.

## Background

Post-traumatic stress disorder (PTSD) is the most widely assessed form of mental distress in cross-cultural studies conducted amongst populations exposed to mass conflict and displacement [1]. Nevertheless, there have been longstanding concerns about the universality of PTSD as a diagnostic category when applied across cultures. One approach to examining this question is to assess whether the same factor structure can be identified in culturally diverse populations as has been described in populations of western societies. We examine this issue based on an analysis of the Harvard Trauma Questionnaire (HTQ) completed by a large community sample in conflict-affected Timor-Leste.

The HTQ is the most widely used measure for assessing PTSD across post-conflict societies of diverse cultural backgrounds [2]. The PTSD symptoms of the HTQ were initially derived from the third edition of the Diagnostic and Statistical Manual (DSM-III) [3], although later studies demonstrated that the items assessed conformed to the three-factor structure (re-experiencing, avoidance/numbing, and arousal) of DSM-IV-TR [4, 5]. The HTQ has been adapted and translated into multiple languages, being applied to conflict-affected and refugee populations from diverse regions of the world, including in Asia [2, 4, 6], the Former Yugoslavia [5, 7], the Middle East [8], and Sub-Saharan Africa [9, 10]. Extensive testing of the measure [5, 7, 11] has supported the validity and reliability of the HTQ PTSD measure in a range of cultures [12].

Establishing the factorial structure of PTSD, even in western populations, has been made more difficult by the serial changes made to the criteria for diagnosing the disorder across successive revisions of the DSM. Although DSM-IV broadly followed the structure of the preceding DSM-III and DSM-III-R by defining three symptom domains of re-experiencing, avoidance/numbing, and arousal [13], in DSM-5, PTSD has undergone a fundamental reformulation with the separation of the numbing and avoidance clusters into two distinct constellations [14].

 A recent systematic review of studies undertaken on a variety of measures of PTSD (not including the HTQ) in high income countries has yielded mixed findings for competing models of PTSD in that there was broad evidence for a four-factor structure Emotional Numbing (EN) as well as a five factor Dysphoric-Arousal (DA) structure, with recent studies suggesting that the DA model demonstrated a good fit in several trauma samples in Anglophone countries [15]. Whereas the EN model is consistent with DSM-5 structure, the DA model comprises five domains of intrusions, avoidance, numbing, anxious arousal (startle response, hypervigilance) and dysphoric-arousal (concentration impairment, irritability, and insomnia) [15]. Remarkably, although previous studies have investigated the factorial structure of PTSD based on the HTQ, none has examined specifically for the aforementioned four- and five-factor models [16–20] amongst culturally diverse populations exposed to mass conflict.

In undertaking a comprehensive analysis of the factorial structure of PTSD based on the HTQ, it is necessary also to consider the proposed ICD-11 formulation of the category, especially because it differs substantially from that of DSM-5, both in the number of symptom domains specified and the symptom composition of each. Derived from the ICD-10 structure, ICD-11 represents PTSD according to the conventional three domains (re-experiencing/intrusions, avoidance, and hyper-arousal), although in the more recent revision, the number of items in each domain has been substantially reduced [21]. Studies undertaken in the USA, most amongst survivors of childhood sexual abuse and mass shooting incidents, have supported both the ICD-10 and the ICD-11 PTSD structures [22]. Although the prevalence of ICD-11 PTSD has been examined amongst samples from Cambodia (ICD-11: 8.1%; DSM-IV: 11.2%) and Columbia (ICD-11: 44.4%; DSM-IV: 55%), no study has investigated the factorial structures of both the ICD-10 and ICD-11 PTSD in a culturally diverse population exposed to mass conflict.

The majority of studies using the HTQ have applied a predefined symptom threshold (2.5) for identifying clinical cases of PTSD, a score derived from the addition of item scores, each rated on a frequency scale of 1 to 4, the sum being divided by the number of items. The conventional 2.5 threshold was identified over two decades ago in convergence studies in which DSM –III based clinical interviews were used to calibrate the HTQ amongst refugees from Southeast Asia. In contrast, our more recent concordance study undertaken in Timor-Leste, in which we calibrated the HTQ against the gold-standard clinical structured interview for DSM-IV disorders, found that a threshold score of 2.2 yielded the best balance between specificity and sensitivity on the former measure [11, 23]. As yet, however, there are no data comparing case assignments based on symptom score cut-offs on the HTQ with formulations of PTSD based on the contemporary DSM or ICD systems.

One method for assessing the concurrent validity of contemporary structures of PTSD is by comparing case assignments of competing formulations with known correlates of the disorder. For example, there is extensive evidence in the post-conflict field that PTSD is associated with female gender, high levels of exposure to trauma and ongoing conditions of adversity [1, 24–26]. In addition, examining which formulation of PTSD is associated with an index of functional impairment offers a further test of the relative validity of each structure. We therefore examine associations of alternative structures of PTSD with these correlates in our present study amongst the Timorese.

As a study site, Timor-Leste offered an opportunity to test aspects of the construct of PTSD in a society that is culturally distinct from high income settings in which most factorial studies have been undertaken. At the time of the study, the population had minimal exposure to western concepts of traumatic stress or other constructs of mental disorder. The population was exposed to high levels of trauma during the prolonged period of conflict during the Indonesian occupation of the territory (1975–1999), a period of low-grade war in which the indigenous population was exposed to atrocities, extrajudicial murders, incarceration and torture [27]. Many Timorese died as a consequence of violence, forced displacement, famine and untreated disease. Following national independence in 2002, a further period of internal conflict (2006–7) resulted in deaths, injuries, burning of houses and internal displacement of communities [11].

The aims of our analysis were to 1) assess the factorial structure of PTSD according to the ICD-10, ICD-11, DSM-IV, four-factor Emotional Numbing (DSM-5 consistent) and five-factor Dysphoric-Arousal models, respectively. In so doing, we note that the range of items in the HTQ precludes a direct examination of the DSM-5 criteria, the four-factor Emotional Numbing model tested herein therefore representing the closest approximation to that structure; 2) compare the prevalence of PTSD assignments based on these criteria and by the conventional HTQ cut-off score; and as a measure of concurrent validity 3) examine PTSD case assignments with established correlates of PTSD including sociodemographic characteristics, trauma count (TC), adversity count (AC), an index of severe psychological distress, and functional impairment.

## Methods

### Sample

The study was conducted between May, 2010 and November, 2011, involving a household survey of all men and women, 18-years and older, residing in two villages in Timor-Leste. The sites were an urban administrative area (*suco*) in Dili, the capital of Timor-Leste, and a rural village located an hour’s drive away. We selected these sites for our earlier survey in 2004 because the Timor-Leste National Directorate of Statistics judged the two resident communities as reflecting the broad range of socio-demographic characteristics of the national population as a whole. Each of the two administrative units is defined by contiguous hamlets (aldeias) under the administration of one chief (chefe). Both locations were directly affected by the longstanding resistance war against the Indonesian occupation and by the subsequent episode of internal conflict that occurred in 2006–7. We used GPS coordinates and aerial maps produced by the Office of Statistics to locate all dwellings in the two locations.

The study was approved by the Human Research Ethics Committee of the University of New South Wales, the Ministry of Health of Timor-Leste, and the chiefs of each village. Participants provided written or witnessed verbal consent.

### Measures

We undertook extensive qualitative and quantitative research, serially field testing and refining mental health measures (with reference to a committee including expatriate and Timorese members) to ensure that the mental health constructs we sought to examine were recognized and regarded as commonly experienced in the community. In the process, we refined items to ensure their cultural, semantic and linguistic appropriateness when translated and applied in Timor-Leste, all interviews being conducted in the lingua franca, Tetum [28].

Posttraumatic stress disorder (PTSD) symptoms and psychological distress.

PTSD symptoms were assessed using the relevant section of the HTQ [2], comprising 16 items scored on a four-point scale (1 = none, 2 = some of the time, 3 = a lot of the time, 4 = most of the time). The adapted HTQ included an additional symptom of physiological reactivity in response to reminders of the trauma, dividing the original single item into the two DSM-IV criteria which differentiate between psychological and physiological reactions to reminders.

To assess psychological distress, we used the Kessler-10 scale, consisting of 10 items indexing depressive but also anxiety and somatic symptoms, each item scored on a five-point scale (1 = not at all, 2 = a little of the time, 3 = some of the time, 4 = most of the time, 5 = all of the time).

Both the PTSD (based on 4-point Likert scale) and Kessler-10 (K10) scales demonstrated high levels of internal reliability (HTQ PTSD, Cronbach’s α = 0.95; K-10, α = 0.92). A convergence study conducted previously amongst a subsample of respondents recruited from the survey, compared the HTQ and K10 with the relevant categories of PTSD and major depressive disorder of the Structured Clinical Interview for the Diagnostic and Statistical Manual IV (SCID) applied in a blinded manner by experienced psychologists [23]. There was a sound level of convergence for both indices: Area Under the Curve (AUC) for PTSD 0.82 (95% CI: 0.71–0.94) and for the K10 0.79 (95% CI: 0.67–0.91). An HTQ score of 2.2 provided the best cut-off for PTSD: sensitivity 77.3%, specificity 87.5%, and correct classification 83%. The dichotomized HTQ item pool showed sound reliability (Kuder-Richardson coefficient/KR20 = 0.83). For the K10, the international cut-off score of 30 or more provided the highest level of convergence: sensitivity 92.3%, specificity 66%, and correct classification 71% [29]. The lower specificity is likely to reflect the inclusion of anxiety and somatic symptoms in addition to depressive symptoms in the K10.

### Exposure to conflict-related traumatic events

We assessed the 23 conflict-related traumatic events (TEs) listed in the HTQ [2], modified to the context of Timor-Leste. TEs were assessed for both the Indonesian occupation (1975–1999), and the subsequent period following national independence which included the episode of internal conflict of 2006–07. Items involved traumas directed at the self and others, including losses and separations. Typical items included political imprisonment, assault, torture, witnessing murder, exposure to atrocities, losses/separations of family or close others, and severe deprivation of medical care for self or others. We generated a composite trauma count (TC) by collapsing responses assessed for both historical periods; an item endorsed for one or both historical periods was assigned a score of 1 whereas a score of 0 indicated no exposure to that event for either of the two historical periods.

### Ongoing adversities

We applied an inventory of ongoing adversities based on community consultations and refinement of items via an iterative process of piloting and feedback. Items included, amongst others, insufficient food, inadequate finances (for school fees, to meet traditional obligations to family), poor shelter, unemployment, and experiences of ongoing conflict (with spouse, children, extended family, young people, and the wider community). Each item was rated on a five-point scale (1 = not a problem, 2 = a bit of a problem, 3 = moderately serious problem, 4 = a serious problem, 4 = a very serious problem). We applied an adversity count (AC) in the present analysis.

### Functional impairment

Functional impairment was assessed using a community-derived index. Prior to the survey, the index was developed based on qualitative data gathered from key informant interviews and two focus groups (comprising men and women 18 to 70 years old) involving chiefs of each village and community members [30]. Participants were asked to rate on a five-point scale (1 = not at all, 2 = little, 3 = moderate, 4 = a lot, 5 = often can’t do task) the level of difficulty they experienced in undertaking or performing activities related to four specific items/domains including domestic duties, working/studying, taking care of family, and socializing. We created a composite index based on an addition of all endorsed functional domains using dichotomized items (0 = none, 1 = little/moderate/extreme difficulties).

### Field personnel training

Eighteen field personnel received two-weeks training followed by 2 months of field testing and piloting of survey measures supervised by expatriate staff. Pairs of interviewers were required to achieve a consistent 100% inter-rater reliability over five interviews on the symptom measures prior to commencing the study. The interviews lasted an hour and were conducted in participants’ homes in a semi-structured format in which questions were read verbatim to participants, most of whom had low literacy, with additional clarifications and explanations provided as needed to ensure full comprehension.

### Statistical analysis

Frequency of endorsement (and percentages) were calculated for individual HTQ symptoms of PTSD. Our preliminary analysis indicated that the responses of HTQ items skewed towards the lower end of the severity spectrum, providing the grounds for dichotomizing scores on statistical ground (0 = not at all/a little and 1 = quite a lot/extremely) [31].

Confirmatory Factor Analysis (CFA) was conducted based on the ICD-10 and the proposed ICD-11 symptom constellations for PTSD as well as for the DSM-IV, the four factor Emotional Numbing and the Dysphoric-Arousal models which approximated the DSM5 structure.

CFA models were estimated using the robust mean- and variance-adjusted Weighted Least Square method (WLSMV), an established statistical procedure recommended for analysing data involving dichotomous variables [31, 32].

We evaluated model fit by using recommended goodness-of-fit and comparative indices, including the chi-square(χ^2^) test, Comparative Fit Index (CFI), Tucker Lewis Index (TLI), and Root Mean Square Error of Approximation (RMSEA). Specifically, a CFI or TLI above 0.95 and a RMSEA below 0.06 indicate a good fit between the model and the data. A moderate fit is indicated by a CFI above 0.90 and a RMSEA below 0.08 [33–36]. Given the large sample size, as indicated by our past modelling analyses [37], we anticipated that good fitting model(s) would have a statistically significant chi-square. In the CFA, we calculated standardized factor loadings and the covariance across factors. In general, a factor coefficient of 0.70 or above is considered to be a reliable indicator of a strongly loaded item; and a cross-factorial correlation of 0.90 or above indicates a high correlation between factors.

We assigned cases in each model based on the appropriate HTQ cut-off score and/or HTQ algorithms, in the latter case based on a mapping of items according to ICD-10, ICD-11 and DSM-IV criteria. The Z-test was used to examine for significant  differences in prevalence of PTSD according to these diagnostic criteria. Cohen’s kappa was calculated to assess the level of diagnostic concordance between PTSD assignments. Finally, a series of logistic regression analyses were conducted to examine associations between PTSD assignments according to ICD-10, ICD-11 and DSM-IV criteria and the clinical threshold of 2.2, with relevant socio-demographic characteristics (model 1); trauma count (TC) and adversity count (AC) (model 2); severe psychological distress and incremental levels of functional impairment (model 3). The analysis was not possible for the four factor models as there were no criteria for assigning caseness in these models. The logistic regression results were expressed as odds ratios with 95% confidence intervals (CI). Analyses were performed using STATA version 13 [38] and Mplus version 7 [32].

## Results

### Socio-demographic characteristics

From the eligible pool of 3597 adults identified in the catchment areas, 2964 completed interviews, a response rate of 82.4% (non-response was due to refusal, and inability of our field staff to make contact in spite of five visits to the dwelling). The analytic sample included 1451 men (49%) and 1513 women (51%) with an overall mean age of 36.4 years.

Table 1 indicates that 62% of the participants resided in rural area and about two thirds (67.9%) were married, a quarter being single/never married (25.5%) and the remainder were widowed or divorced/separated. About 23.9% had completed junior school, 26.3% senior high school, and 10.7% had received post-school education (college/university). A third (34%) were engaged in paid employment (in a range of work including government and private sectors), 35% were unemployed and 6.1% retired; the remainder were involved in subsistence farming/ domestic duties or were unable to work because of physical disability.

**Table 1:** Socio-demographic characteristics and mental health characteristics of the sample (*n* = 2964)

Socio-demographic characteristics and mental health measures	Number of respondents (*n* = 2964)	% of total
Sex: Female	1451	49.0
Male	1513	51.1
Location: Rural	1844	62.0
Urban	2013	67.9
Age group (years): <24	578	19.5
25–34	1017	34.3
35–44	632	21.3
45–54	324	10.9
≥ 55	413	13.9
*Mean age, year (sd)*	*36.4 (14.4)*	
Marital status: Married	2013	67.9
Single/never married	756	25.5
Widowed	171	5.8
Divorced/Separated	24	0.8
Educational attainment: Completed primary	343	11.6
Completed junior high school	364	12.3
Completed senior high school	779	26.3
Completed tertiary	317	10.7
Employment: Retired	180	6.1
Unable to work due to physical disability	43	1.5
Unemployed	1035	34.9
Employed (government/private sectors)	1032	34.0
Subsistence farming	359	12.1
Domestic duties	315	10.6
Mental health outcomes		
PTSD (2.2 threshold)	453	15.3
Severe psychological distress (K10 ≥ 30)	447	15.1
Functional impairment		
1 domain of impairment	96	3.2
2 domains of impairment	66	2.2
3 domains of impairment	183	6.2
4 domains of impairment	2442	82.4

### Threshold scores for symptoms of PTSD severe psychological distress and functional impairment

One in seven (*n* = 453; 15.3%) met criteria for PTSD based on the clinical HTQ threshold (≥2.2); 15.1% (*n* = 447) reported severe psychological distress (K10 ≥ 30), and 82.4% (*n* = 2442) reported difficulties in at least 1 domain of functioning including performing domestic duties, attending school, going to work, attending to the needs of family members, and socializing with others (Table 1).

### Confirmatory factor analysis (CFA)

Table 2 maps the constituent items of the HTQ based on the ICD-10, the proposed ICD-11, the DSM-IV, the four factor Emotional Numbing and Dysphoric-Arousal models. Standardized factor loadings for all models tested are presented in Table 3. A good fit was achieved for the three-factor models based on ICD-10 (χ^2^ (62 df) = 688.59, *P* ≤ 0.001, CFI = 0.93, TLI = 0.91, RMSEA = 0.058) and ICD-11 (χ^2^ (6 df) =34.16, *P* ≤ 0.001, CFI = 0.99, TLI = 0.98, RMSEA = 0.04). The DSM-IV three-factor model (χ^2^ (116 df) =981.14, *P* ≤ 0.001, CFI = 0.93, TLI = 0.92, RMSEA = 0.05), the four-factor Emotional Numbing (χ^2^ (113 df) = 995.95, *P* ≤ 0.001, CFI = 0.93, TLI = 0.92, RMSEA = 0.051), and the five-factor Dysphoric-Arousal (χ^2^ (109 df) = 964.93, *P* ≤ 0.001, CFI = 0.93, TLI = 0.91, RMSEA = 0.05) models each produced moderately good fitting solutions. Table 4 reports the goodness of fit statistics for the sequence of CFA models based on the HTQ PTSD symptom list. Standardized factor loadings for all models tested are presented in Table 3.

**Table 2:** Mapping items of the Harvard Trauma Questionnaire (HTQ) based on the ICD-10, ICD-11, DSM-IV, four-factor Emotional Numbing, and five-factor Dysphoric-Arousal models

Symptom Cluster	Symptoms	Item	Corresponding item	Number (*n* = 2964)	%	ICD-10	ICD-11	DSM-IV	EN model	DA model
Intrusion	Intrusive thoughts, flashbacks, disturbing dreams	1	Recurrent thoughts or memories of the most hurtful or terrifying events	463	15.6	I	-	I	I	I
		2	Feeling as though the event is happening again	194	6.5	I	I	I	I	I
		3	Recurrent nightmares	163	5.5	I	I	I	I	I
	Physical/psychological reactions to reminders of trauma	16	Psychological distress when reminded of trauma	176	5.9	I	-	I	I	I
		17	Physiological reactivity to reminders of traumatic event	141	4.8	I	-	I	I	I
Avoidance	Internal avoidance	11	Avoiding activities that remind you of the traumatic or hurtful event.	198	6.7	A	A	AN	A	A
	External avoidance	15	Avoiding thoughts or feelings associated with traumatic or hurtful event	170	5.7	A	A	AN	A	A
Numbing	Diminished interest	4	Feeling detached or withdrawn from people	260	8.8	-	-	AN	N	N
		5	Unable to show emotions	181	6.1	-	-	AN	N	N
		12	Inability to remember parts of the most hurtful or traumatic events	124	4.2	H	-	AN	N	N
		13	Less interest in daily activities	247	8.3	-	-	AN	N	N
	Foreshortened future	14	Feeling as if you do not have a future	383	12.9	-	-	AN	N	N
Hyperarousal	Anxious arousal	6	Feeling jumpy or easily startled	622	21.0	H	H	H	H	AA
		9	Feeling on guard	617	20.8	H	H	H	H	AA
	Dysphoric Arousal	7	Difficulty in concentrating	403	13.6	H	-	H	H	DA
		8	Trouble sleeping	621	21.0	H	-	H	H	DA
		10	Feeling irritable or having outbursts of anger	410	13.8	H	-	H	H	DA

*Abbreviations: I* Intrusion, *A* Avoidance, *N* numbing, *AN* Avoidance/Numbing, *H* hyperarousal, *AA* Anxious Arousal, *DA* Dysphoric Arousal

**Table 3:** Standardised factor loadings for ICD-10, ICD-11, DSM-IV, four-factor Emotional Numbing, and five-factor Dysphoric-Arousal models

	HTQ items	ICD-10			ICD-11			DSM-IV			4-factor Emotional Numbing model				5-factor Dysphoric-Arousal model				
		Intrusion	Avoidance	Hyper-arousal	Intrusion	Avoidance	Hyper-arousal	Intrusion	Avoidance Numbing	Hyper-arousal	Intrusion	Avoidance	Numbing	Hyper-arousal	Intrusion	Avoidance	NumBing	Anxious Arousal	Dysphoric Arousal
1	Recurring thoughts	0.62	-	-	-	-		0.61	-	-	0.61	-	-	-	0.61	-	-	-	-
2	Flashbacks	0.78	-	-	0.78			0.77	-	-	0.77	-	-	-	0.77	-	-	-	-
3	Nightmares	0.62	-	-	0.65			0.71	-	-	0.71	-	-	-	0.71	-	-	-	-
16	Psychological distress to reminders of trauma	0.83	-	-	-			0.80	-	-	0.80	-	-	-	0.80	-	-	-	-
17	Physiological distress to reminders of trauma	0.77	-	-	-			0.75	-	-	0.75	-	-	-	0.75	-	-	-	-
11	External avoidance	-	0.75		-	0.78	-	-	0.71	-	-	0.76	-	-	-	0.76	-		-
15	Internal avoidance	-	0.78		-	0.75	-	-	0.72	-	-	0.77	-	-	-	0.77	-		-
4	Detachment	-	-	-	-	-	-	-	0.75	-	-	-	0.76	-	-		0.76	-	-
5	Restricted affect	-	-	-	-	-	-	-	0.74	-	-	-	0.75	-	-		0.75	-	-
12	Amnesia	-	-	-	-	-	-	-	0.69	-	-	-	0.69	-	-		0.69	-	-
13	Anhedonia	-	-	-	-	-	-	-	0.70	-	-	-	0.71	-	-		0.71	-	-
14	Foreshortened future	-	-	-	-	-	-	-	0.72	-	-	-	0.72	-	-		0.72	-	-
6	Startle response	-	-	0.85	-	-	0.84	-	-	0.84	-	-		0.84	-		-	-	0.87
7	Concentration difficulties	-	-	0.72	-	-	-	-	-	0.73	-	-	-	0.73	-		-	0.69	-
8	Insomnia	-	-	0.73	-	-	-	-	-	0.76	-	-	-	0.76	-		-	0.73	-
9	Hyper-vigilance	-	-	0.85	-	-	0.91	-	-	0.85	-	-	-	0.85	-		-	-	0.88
10	Irritability	-	-	0.68	-	-	-	-	-	0.71	-	-	-	0.71	-		-	0.68	-

**Table 4:** Model Fit indices for tested confirmatory factor analysis (CFA) models

Models	*Χ* ^2^	*df*	CFI	TLI	RMSEA
ICD-10 three-factor model	688.59*, **	62	0.93	0.91	0.058
ICD-11 three-factor model	34.16*, **	6	0.99	0.98	0.040
Three-factor DSM-IV model	981.14*, **	116	0.93	0.92	0.050
Four-factor Emotional Numbing model	995.95*, **	113	0.93	0.92	0.051
Five-factor Dysphoric-Arousal model	964.93*, **	109	0.93	0.91	0.051

*Χ* ^*2*^ Chi-square goodness of fit statistic, *df* degrees of freedom

*CFI* Comparative Fit Index, *TLI* Tucker Lewis Index

*RMSEA* Root-mean-square error of approximation

*Models are significant  at *p* < 0.05; **Models are significant at *p* < 0.01

### Prevalence estimates of ICD-10, ICD-11, and DSM-IV PTSD based on symptom criteria

PTSD symptom criteria were met by 46.2% (*n* = 1369) of the sample for ICD-10, 33.7% (*n* = 998) for ICD-11 and 38% (*n* = 1126) for DSM-IV criteria (data not shown). Although comparisons of each classification with another showed differences that in some instances were statistically significant (Table 5), there was a substantial level of agreement across systems in general, specifically between DSM-IV and respectively, the ICD-10 (kappa = 0.83, Z-score = 45.8, *P* < 0.001) and ICD-11 assignments (kappa = 0.79, Z-score = 43.1), and between ICD-10 and ICD-11 assignments (kappa = 0.74, Z-score = 41.9, *P* < 0.001). Moderate agreement was found between the ICD-11 and HTQ clinical threshold assignments (kappa = 0.51, Z-score = 31.7, *P* < 0.0001). In contrast, low agreement was found between the ICD-10 and HTQ clinical threshold case assignment (kappa = 0.35, Z-score = 69.1).

**Table 5:** Percentage of agreement (kappa) across symptom case assignments derived from the DSM-IV, ICD-10, the proposed ICD-11 PTSD criteria, and the community threshold

PTSD assignments	ICD-10	ICD-11	DSM-IV	2.2 threshold
	% of agreement (kappa)	% of agreement (kappa)	% of agreement (kappa)	% of agreement (kappa)
ICD-10	-	87.5 (0.74)	91.7 (0.83)	69.1 (0.35)
ICD-11	87.5 (0.74)	-	90.2 (0.79)	81.1 (0.51)
DSM-IV	91.7 (0.83)	90.2 (0.79)	-	77.3 (0.46)
2.2 threshold	69.1 (0.35)	81.1 (0.51)	77.3 (0.46)	-

All the kappa coefficients are significant at *p* < 0.001

### Assessment of concurrent validity

Multivariate logistic regression analyses were applied to examine associations of PTSD models with sociodemographic variables (model 1); trauma count and adversity count (model 2); and severe psychological distress (K10 > 30) and functional impairment (model 3). Adjusted odds ratios with 95% CIs are presented in Table 6.

**Table 6:** Logistic regression analyses of socio-demographic (Model 1), psychosocial (Model 2), and mental health predictors (Model 3) of positive PTSD assignments based on the ICD-10, ICD-11, DSM-IV CFA models and the HTQ 2.2 community threshold (PTSD ≥ 2.2)

Socio-demographic and mental health measures	ICD-10	ICD-11	DSM-IV	PTSD (2.2)
	OR (95% CI)	OR (95% CI)	OR (95% CI)	OR (95% CI)
Model 1				
Sex: Female (ref.: male.)	1.55(1.31–1.84)**	1.86(1.55–2.22)**	1.80(1.51–2.15)**	2.36(1.85–3.01)**
Location: Rural (ref: urban.)	0.95(0.79–1.14)	1.01(0.83–1.27)	0.86(0.71–1.04)	1.26(0.96–1.65)
Age group (years): <24 (ref.)	1.0	1.0	1.0	1.0
25–34	1.34(1.04–1.72)*	1.30(0.98–1.71)	1.20(0.92–1.56)	0.95(0.66–1.38)
35–44	1.02(0.76–1.37)	0.89(0.65–1.23)	0.89(0.66–1.21)	0.90(0.59–1.37)
45–54	0.85(0.60–1.21)	0.73(0.50–1.06)	0.69(0.48–0.99)	0.82(0.50–1.32)
≥ 55	0.75(0.53–1.05)	0.69(0.48–0.99)*	0.58(0.40–0.83)**	0.53(0.32–0.87)**
Married (ref: single)	0.97(0.80–1.18)	0.89(0.72–1.09)	0.99(0.82–1.22)	0.73(0.56–0.96)*
Educational attainment: No education (ref.)	1.0	1.0	1.0	1.0
Completed primary	1.12(0.86–1.46)	1.05(0.79–1.40)	1.02(0.78–1.34)	0.96(0.66–1.38)
Completed junior high school	1.17(0.87–1.56)	0.98(0.72–1.34)	1.16(0.86–1.56)	1.07(0.72–1.60)
Completed senior high school	0.87(0.69–1.11)	0.94(0.73–1.22)	0.96(0.75–1.23)	1.12(0.80–1.55)
Completed tertiary	0.73(0.54–1.01)	0.79(0.56–1.12)	0.67(0.47–0.93)*	0.76(0.47–1.42)
Employment: Employed (ref.)	1.0	1.0	1.0	1.0
Engaged in subsistence farming	0.91(0.76–1.09)	0.78(0.64–0.95)*	0.77(0.63–0.93)**	0.66(0.51–0.85)**
Unemployed	1.00(0.60–1.67)	0.85(0.50–1.43)	1.09(0.64–1.82)	0.61(0.31–1.18)
Model 2				
Adversity count (continuous)	1.06(1.03–1.08)**	1.09(1.06–1.12)**	1.07(1.04–1.10)**	1.12(1.08–1.15)**
Trauma count (continuous)	1.15(1.12–1.18)**	1.17(1.14–1.21)**	1.18(1.14–1.21)**	1.22(1.17–1.27)**
Model 3				
Psychological distress: K10 ≥ 30 (ref: K10 < 30)	2.83(2.29–3.52)**	3.07(2.49–3.78)**	3.14(2.55–3.88)**	2.91(2.30–3.68)**
Functional impairment				
No domain of impairment	1.0	1.0	1.0	1.0
1–2 domains of impairment	1.22(0.77–1.95)	1.09(0.58–2.04)	1.22(0.75–2.01)	1.07(0.21–5.41)
3 domains of impairment	1.93(1.24–3.00)**	2.01(1.15–3.52)*	1.27(0.79–2.04)	4.70(1.33–16.61)*
4 domains of impairment	2.10(1.50–2.94)**	3.55(2.27–5.57)**	1.93(1.35–2.77)**	10.97(3.48–34.57)*

*Adjusted odds ratios (ORs) are significant at *p* < 0.05; ** ORs are significant at *p* < 0.01;

*ref.* Indicates used as reference category in logistic regression analysis

Findings revealed that as compared to men, women were more likely to meet symptom criteria for PTSD according to ICD-10 (OR = 1.55, CI: 1.31–1.84), ICD-11 (OR = 1.86, CI: 1.55–2.22), DSM-IV (OR = 1.80, CI: 1.51–2.15), and the HTQ clinical threshold (OR = 2.36, CI: 1.85–3.01). We note that age, occupational and residency (urban/rural) are likely to be context specific so that the associations shown with different categorizations of PTSD may not have general significance and are not emphasized here.

Trauma count and adversity count all showed associations with ICD-10, ICD-11, DSM-4, and the clinical threshold PTSD assignments. In relation to severe psychological distress and functional impairment, we found a dose-response association with all four classification methods, that is positive case assignment for PTSD categorizations based on ICD-10, ICD-11, DSM-IV and the HTQ clinical threshold assignments were associated statistically with psychological distress and functional impairment, respectively (Table 6).

The adjusted odds ratios (ORs) were largest where the highest level of impairment was reported in all four domains ranging from 1.93 (95%CI: 1.35–2.77) for the DSM-IV assignment to 10.97 (95%CI: 3.48–34.57) for the HTQ clinical threshold assignment (Table 6).

## Discussion

Our study is unique in exploring the ICD-10, proposed ICD-11, DSM-IV, four-factor Emotional Numbing and five-factor Dysphoric-Arousal PTSD structures for PTSD according to the HTQ in a culturally distinct population, in this instance, based on a large sample in post-conflict Timor-Leste. Our CFA results found support for all contemporary PTSD factorial models in this population with only marginal differences between them, a result that is consistent with other inquires undertaken amongst Anglophone populations in developed countries. Consistent with the literature and providing support for the concurrent validity of our findings, we found statistical associations between all PTSD models and gender (women reporting a higher prevalence) [39], the quantum of trauma exposure, an index of ongoing adversity, a measure of severe psychological distress, and levels of functional impairment. Notably, however, the prevalence rates of PTSD showed marked variation across the models, with a greater number of persons meeting ICD-10, ICD-11 and DSM-IV criteria compared to those who reached the clinical HTQ threshold.

The strengths of our study include the large sample, the careful approach to recruitment, and high response rate (82.4%). The restriction of our sample to two villages means that further studies will be needed to test the generalizability of our findings to populations in Timor and wider afield. In spite of our systematic approach in adapting and translating our measures [40], we cannot discount the risk of transcultural errors in assessment. Although anamnestic bias can lead to inaccuracy in recording trauma and losses, we note that, in general, the events recorded are consistent with the known history of conflict in Timor-Leste. The culturally adapted HTQ included symptoms based on the DSM-IV-TR, thereby precluding a direct examination of the DSM-5 criteria, the four-factor Emotional Numbing model we tested representing the closest approximation to that structure. The clinical threshold of 2.2 we applied to generate PTSD caseness was based on our clinical calibration of the measure compared with the structured clinical interview for DSM-IV disorders [23]. This finding illustrates the need to redefine the threshold in each setting in that our cutoff differed from the HTO cutoff established in other contexts [4, 8, 10]. Finally, our analysis was restricted to the symptom domains of PTSD given that the HTQ is not designed to assess all aspects of caseness, in particular, associated functional impairment.

These caveats notwithstanding, our key findings, based on the sequence of CFAs conducted, provide support for the capacity of the HTQ to assess PTSD symptoms in this transcultural setting. The measure was found to yield findings consistent with a range of established PTSD factorial models, including the ICD-10, the proposed ICD-11, the DSM-IV, four-factor Emotional Numbing (DSM-5 consistent) and the five-factor Dysphoric-Arousal models of PTSD, an important finding in the transcultural field. The only broadly relevant study in the field was one that found support for the Dysphoric-Arousal model [15] amongst a clinic sample of Arabic speaking refugees undergoing psychiatric treatment in Denmark [41]. With the exception of our study amongst refugees from West Papua [42], no studies have tested the ICD-10 or the proposed ICD-11 PTSD structure in a large post-conflict population. Our findings therefore add further evidence in support of the factorial structures of a range of PTSD classification systems including the ICD-10, ICD-11, DSM-IV, four-factor Emotional Numbing and the five-factor Dysphoric-Arousal models. The finding that all PTSD structures tested in this population provided similarly adequate solutions accords with a recent systematic review of the contemporary PTSD structures in which the current body of studies provided support for both the EN and DA models (noting that the former corresponds directly to the DSM-5 structure) across diverse trauma samples from western countries. Together, the findings provide strong evidence for the separation of numbing and arousal symptoms into two distinct constellations as formulated in DSM-5. In deriving these conclusions, it is important to recognize that CFA allows assessment of the correspondence between the constituent items and their respective domains, only one source of evidence to determine which cluster symptoms are most appropriate to making a clinical diagnosis. Hence a range of studies using various methodologies is needed in order to determine more clearly what group of symptoms best represents a universal constellation of PTSD at a universal level.

Our demonstration of a dose-response relationship between trauma exposure, ongoing adversity and PTSD as assessed by all the models tested is consistent with a well-established association in the post-conflict and refugee field [43]. In addition, logistic regression analysis found that all models of PTSD symptoms were associated with severe psychological distress and incremental levels of functional impairment (in 3 and 4 functional domains), a finding that accords with the post-conflict mental health literature in general [44, 45]. This anticipated pattern of correlates of PTSD across all methods of categorization provides further support for the construct validity of PTSD and the use of the HTQ as a screening measure in this transcultural population. Together, our findings suggest that the adapted HTQ may have ongoing utility in capturing the contemporary construct of PTSD and hence can be used validly as a screening and monitoring instrument in the Timorese population as a whole.

## Conclusions

Our study found considerable support for the ICD-10, ICD-11, DSM-IV, four-factor Emotional Numbing (consistent with the DSM-5 formulation of PTSD) and Dysphoric-Arousal PTSD structures in a large conflict-affected population in Timor-Leste. Case assignments using various models showed consistent associations with female gender, trauma exposure, ongoing adversity, severe distress, and functional impairment, providing evidence of concurrent validity of the HTQ symptom measure. Although symptom prevalence estimates varied across classifications, there was adequate agreement in PTSD assignments across the systems. Together, the data suggest that the HTQ represents a robust measure for assessing PTSD symptoms across several models of the disorder, adding to the growing body of evidence supporting the utility of the measure in the transcultural setting.

## Abbreviations

A: Avoidance
AA: Anxious Arousal
AN: Avoidance/Numbing
CFA: Confirmatory factor analysis
CFI: Comparative Fit Index
DA: Dysphoric Arousal
DSM: Diagnostic and Statistical Manual
H: Hyperarousal
HTQ: Harvard Trauma Questionnaire
I: Intrusion
ICD: International Classification of Diseases
N: Numbing
PTSD: Posttraumatic stress disorder
RMSEA: Root Mean Square Error of Approximation
TLI: Tucker Lewis Indhex
